# Magnetic Structure Determinations at NBS/NIST

**DOI:** 10.6028/jres.106.047

**Published:** 2001-12-01

**Authors:** J. W. Lynn, J. A. Borchers, Q. Huang, A. Santoro, R. W. Erwin

**Affiliations:** National Institute of Standards and Technology, Gaithersburg, MD 20899-0001

**Keywords:** applied magnetic field, magnetic multilayers, magnetic order parameter, magnetic structure, magnetic superconductors, magnetic symmetry, neutron diffraction, polarization analysis, pressure dependence

## Abstract

Magnetic neutron scattering plays a central role in determining and understanding the microscopic properties of a vast variety of magnetic systems, from the fundamental nature, symmetry, and dynamics of magnetically ordered materials to elucidating the magnetic characteristics essential in technological applications. From the early days of neutron scattering measurements at NBS/NIST, magnetic diffraction studies have been a central theme involving many universities, industrial and government labs from around the United States and worldwide. Such measurements have been used to determine the spatial arrangement and directions of the atomic magnetic moments, the atomic magnetization density of the individual atoms in the material, and the value of the ordered moments as a function of thermodynamic parameters such as temperature, pressure, and applied magnetic field. These types of measurements have been carried out on single crystals, powders, thin films, and artificially grown multilayers, and often the information collected can be obtained by no other experimental technique. This article presents, in an historical perspective, a few examples of work carried out at the NIST Center for Neutron Research (NCNR), and discusses the key role that the Center can expect to play in future magnetism research.

## 1. Introduction

There have been hundreds of studies of magnetic structures and magnetic ordering at the NCNR, on wide classes of materials. A comprehensive review of this work is not possible within this context, so in the current article we simply discuss a few examples of the type of work that has been carried out at the NCNR, and provide some additional representative references to the wider distribution of work. The neutron instrumentation required to make such measurements is generally the same as needed for the determination of crystallographic structures on a variety of length scales, and the history of the available instrumentation is discussed elsewhere in this volume. Here we briefly note the neutron instrumentation presently available to the magnetism community at the NCNR, and mention plans for new instrumentation which will take the field in the United States into the next decade and beyond.

Magnetic neutron scattering originates from the interaction of the neutron’s spin with the unpaired electrons in the sample. The strength of this magnetic dipole-dipole interaction is comparable to the neutron-nuclear interaction, and thus there are magnetic cross-sections that are analogous to the nuclear ones that reveal the complete structure and full range of lattice dynamics of materials over wide ranges of length scale and energy. The traditional role of magnetic neutron scattering is the measurement of magnetic Bragg intensities in the magnetically ordered regime, which can be used to determine the spin configuration and directions of the atomic magnetic moments as a function of temperature, pressure, and applied magnetic field, on single crystals samples, powders, thin films and artificially grown multilayers [1]. Early studies addressed materials such as spinels and ferrites, followed by rare-earth intermetallics [2] and rare earth hydrides [3]. One topic that has sustained interest over the years, though, is the magnetic ordering that occurs in superconductors [4–7], and we will present some examples below. Other types of systems that have been investigated with magnetic diffraction include heavy fermion systems [8–13], ruthenates [14–15] and cobalates [16–17], amorphous [18] and nanocrystalline [19–21] systems, frustrated magnets [22–24], molecular magnets, [25–26] and colossal magnetoresistive oxides [27–33].

For magnetic phenomena that occur over length scales that are large compared to atomic distances, the technique of magnetic Small Angle Neutron Scattering (SANS) can be applied, in analogy to structural SANS. This is an ideal technique to explore domain structures [33], ferromagnetic correlations [34] and long wavelength oscillatory magnetic states in superconductors [35–36], vortex structures in superconductors [37–39], and other spatial variations of the magnetization density on length scales from 1 nm to 1000 nm. Another specialized technique is neutron reflectometry, which can be used to investigate the magnetization profile in the near-surface regime of single crystals [40–41], as well as the magnetization density of thin films and multilayers [42–60], in analogy with structural reflectometry techniques. Reflectometry has enjoyed dramatic growth during the last decade due to the rapid advancement of atomic deposition capabilities.

There has been a natural evolution in the complexity of materials that have been investigated; early work tended to be on relatively simple systems, but as the instrumentation has improved and calculational capabilities have expanded, ever more complex structures have been successfully tackled. For the colossal magnetoresistive materials of current interest, for example, the lattice, electronic, and magnetic degrees of freedom are intertwined, requiring that the crystal and magnetic structures be solved together.

## 2. Magnetic Diffraction

The integrated intensity for a magnetic Bragg reflection is given (for a simple collinear magnetic structure) by [61]

$$I_{M}=CM_{\tau }A\left(\theta \right)\left(\frac{\gamma e^{2}}{2mc^{2}}\right)\langle 1-{\left(\tau \cdot M\right)}^{2}\rangle {\left|F_{M}\right|}^{2}$$

where the neutron-electron coupling constant in parenthesis is −0.27 × 10^−12^ cm, ***τ*** and ***M*** are unit vectors in the direction of the reciprocal lattice vector ***τ*** and the spin direction, respectively, and the orientation factor <1−(***τ*** · ***M***)^2^> must be calculated for all possible domains. *C* is an instrumental constant which includes the resolution of the measurement, *A*(*θ*) is an angular factor which depends on the method of measurement, and *M_τ_* is the multiplicity of the reflection (for powders). The magnetic structure factor *F*_M_ is given by

$$F_{M}=\sum_{j=1}^{N}{\langle \mu_{z}\rangle }_{j}f_{j}\left(\tau \right)e^{-W_{j}}e^{i\tau \cdot r_{j}}$$

where 〈*μ_z_*〉*_j_* is the thermal average of the aligned magnetic moment of the magnetic ion at the *j*th site at position ***r****_j_*, ***W****_j_* is the Debye-Waller factor for the *j*th atom, *f_j_*(***τ***) is the magnetic form factor (Fourier transform of the magnetization density), and the sum extends over all magnetic atoms in the unit cell. We see from these expressions that neutrons can be used to determine several important quantities [1]; the location of magnetic atoms and the spatial distribution of their magnetic electrons; the temperature, field, and pressure dependence of 〈*μ_z_*〉, which is directly related to the order parameter for the phase transition (e.g., sublattice magnetization). The preferred magnetic axis $\left(\hat{M}\right)$ also can often be determined from the relative intensities. Finally, the scattering can be put on an absolute scale by internal comparison with the nuclear Bragg intensities from the same sample, whereby the saturated value of the magnetic moment can be obtained.

**Fig. 1:**
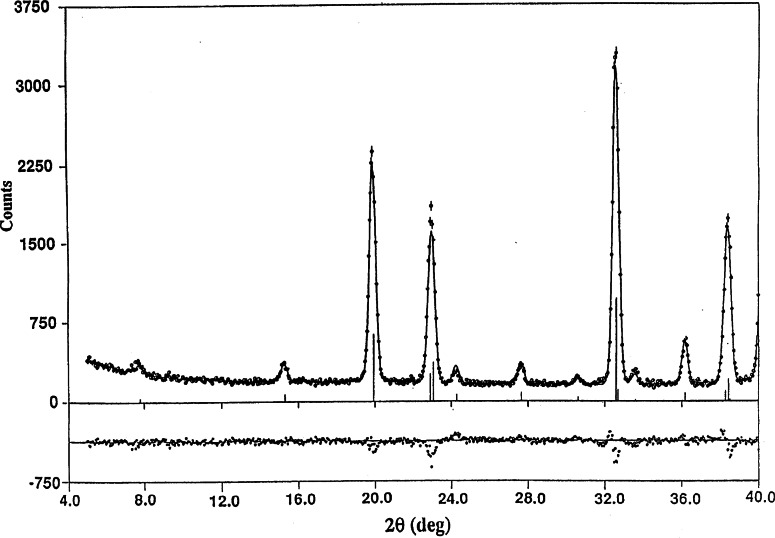
Calculated (solid curve) and observed intensities for a powder of YBa_2_Fe_3_O_8_. The differences between calculated and observed intensities are shown at the bottom [62].

**Fig. 2:**
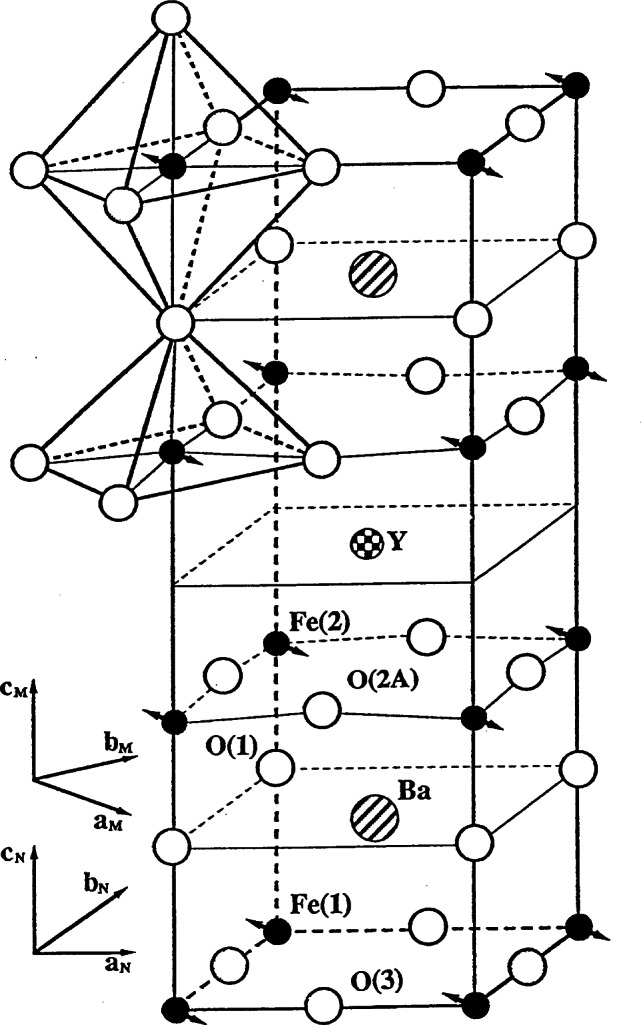
Crystal and magnetic structure for YBa_2_Fe_3_O_8_ [62].

As an example, a portion of the powder diffraction pattern from a sample of YBa_2_Fe_3_O_8_ is shown in Fig. 1 [62–64]. The solid curve is a profile refinement of both the antiferromagnetic and crystallographic structure for the sample, and the experimental intensities are indicated by the open circles. The error bars indicated for the data points are statistical uncertainties that represent one standard deviation, and this notation is followed throughout this article. From these data we can determine the crystal structure, lattice parameters, site occupancies, etc., as well as the magnetic structure and value of the ordered moment. The results of the analysis are shown in Fig. 2; the crystal structure is identical to the structure for the 1–2–3 superconductor with the Fe replacing the Cu, and the magnetic structure is also the same as has been observed for the Cu spins in oxygen-reduced (semiconducting) material [5].

**Fig. 3:**
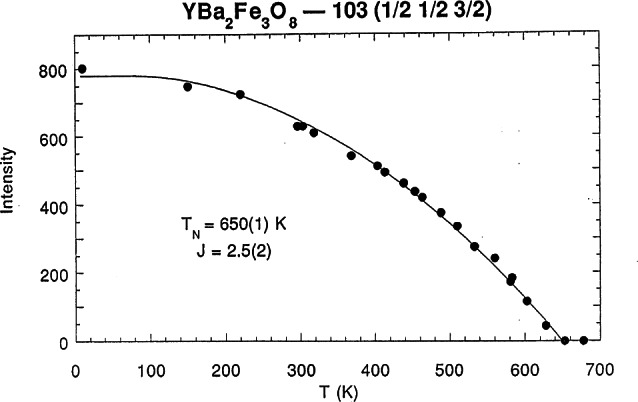
Temperature dependence of the intensity of the magnetic reflection [63].

**Fig. 4:**
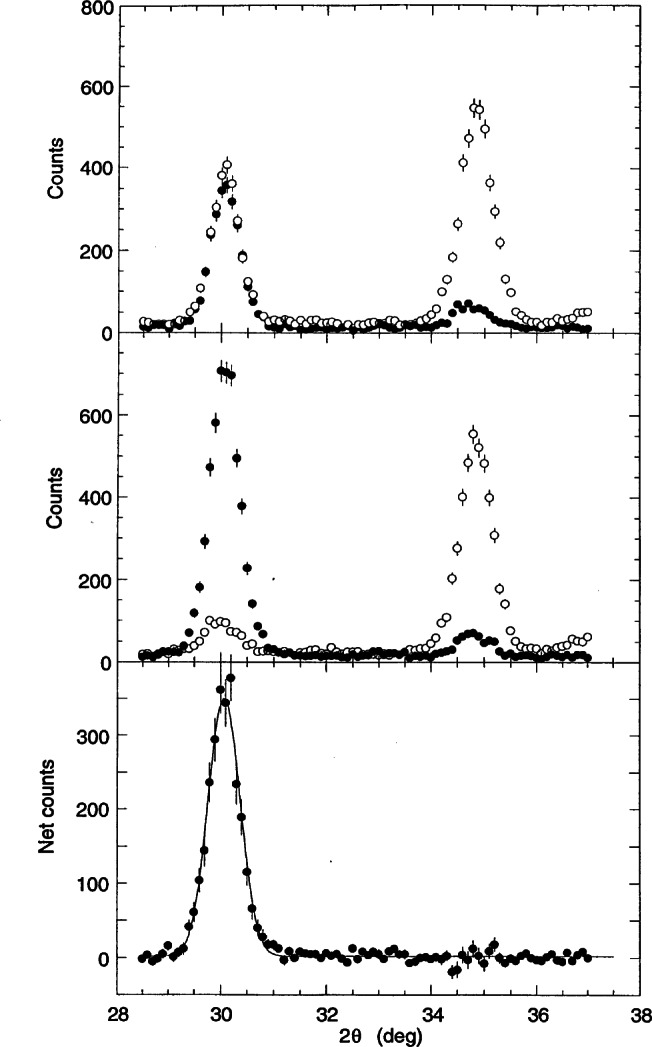
Polarized neutron scattering. The top portion of the figure is for ***P***⊥***τ***, where the open circles show the non-spin-flip scattering and the filled circles are in the spin-flip configuration. The low angle peak has equal intensity for both cross sections, and thus is identified as a pure magnetic reflection, while the ratio of the (+ +) to (− +) scattering for the high angle peak is just the instrumental flipping ratio. Hence this is a pure nuclear reflection. The center portion of the figure is for ***P***║***τ***, and the bottom portion is the subtraction of the spin-flip data for the ***P***⊥***τ*** configuration from the spin-flip data for ***P***║***τ***. Note that in the subtraction procedure all background and nuclear cross sections cancel, isolating the magnetic scattering [62].

Figure 3 shows the temperature dependence of the intensity of one of the magnetic peaks, which clearly reveals a phase transition at 650 K. To establish that this scattering is purely magnetic in origin, and in particular that there is no crystallographic distortion related to a substantial magnetoelastic interaction, the neutron polarization technique was used to unambiguously identify and separate the magnetic and nuclear scattering. The scattering for a nuclear Bragg peak always preserves the spin alignment of the neutron (non-spin-flip scattering), while the magnetic cross sections depend on the relative orientation of the neutron polarization ***P*** and the reciprocal lattice vector ***τ***. In the configuration where ***P***⊥***τ***, half the magnetic Bragg scattering involves a reversal of the neutron spin (denoted by the (− +) configuration), and half does not, and for a purely magnetic reflection the spin-flip (− +) and non-spinflip (+ +) intensities should then be equal in intensity. For the case where ***P***║***τ***, all the magnetic scattering is spin-flip. Figure 4 shows the polarized beam results for two peaks, at scattering angles (for this wavelength) of 30° and 35°; these correspond to the peaks at 19.5° and 23° in Fig. 1. The top section of the figure shows the data for the ***P***⊥***τ*** configuration. The peak at 30° has the identical intensity for both spin-flip and non-spin-flip scattering, and hence we conclude that this scattering is purely magnetic in origin as inferred from Fig. 3. The peak at 35°, on the other hand, has strong intensity for (+ +), while the intensity for (− +) is smaller by the instrumental flipping ratio. Hence this peak is a pure nuclear reflection. The center row shows the same peaks for the ***P***║***τ*** configuration, while the bottom row shows the subtraction of the ***P***⊥***τ*** spin-flip scattering from the ***P***║***τ*** spin-flip scattering. In this subtraction procedure instrumental background, as well as all nuclear scattering cross sections, cancel, isolating the magnetic scattering. We see that there is magnetic intensity only for the low angle position, while no intensity survives the subtraction at the 35° peak position. These data unambiguously establish that the 30° peak is purely magnetic, while the 35° peak is purely nuclear. This simple example demonstrates how the technique works; obviously it plays a more critical role in cases where it is not clear from other means what is the origin of the peaks, such as in regimes where the magnetic and nuclear peaks overlap, or in situations where the magnetic transition is accompanied by a structural distortion.

## 3. Magnetic Superconductors

The effects of magnetic impurities and the possibility of magnetic ordering in superconductors have had a rich and interesting history, and neutrons have played an essential role in determining the nature of the magnetic order since the Meissner screening of the superconducting electrons masks the magnetism from most probes. Early work was on the (*R*-Ce)Ru_2_ (*R* = rare earth ion) substitutional alloys [34], where strong ferromagnetic correlations were found to coexist with superconductivity, but the first examples of true long range magnetic order coexisting with superconductivity were provided by the ternary Chevrel-phase superconductors (*R*Mo_6_S_8_) [35–36] and related *R*Rh_4_B_4_ compounds [65]. The magnetic ordering temperatures were all low, ≈1 K, and thus it was argued that electromagnetic (dipolar) interactions should dominate the energetics of the magnetic system. For most materials antiferromagnetism is favored, and the magnetization averages to zero on the length scale of a unit cell, resulting in a weak influence on the superconducting state. The next class of materials that were investigated were the Heusler alloy series *R*Pd_2_Sn, [66–67] followed quickly by the cuprate superconductors (e.g., *R*Ba_2_Cu_3_O_6+x_) which offer new and interesting perspectives into our understanding of “magnetic superconductors” [68–85]. The rare earth ions order at low temperature similar to “conventional” magnetic superconductors [77–85], while in the de-oxygenated, insulating state the Cu spins order above room temperature [68–77]. Both types of spins exhibit low-dimensional behavior [6]. In the superconducting state the rare-earth spins still order magnetically, as for example in ErBa_2_Cu_3_O_7_, where the Er moments exhibit two dimensional behavior [78–79], and it turns out to be an ideal two-dimensional S = 1/2 Ising antiferromagnet. More recently, the magnetic ordering has been investigated in the single-layer electron doped superconductors (such as Sm_2_CuO_4_ [85]) and the *R*Ni_2_B_2_C class of superconductors [86–88], where the magnetic ordering temperatures are much too high to be explained by dipolar interactions and there is a clear competition with the superconductivity.

**Fig. 5:**
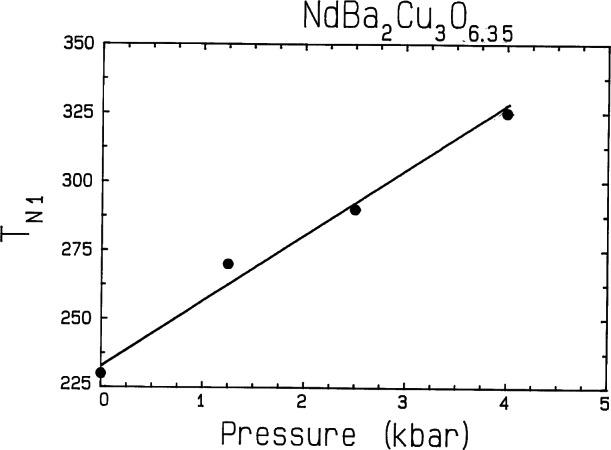
Pressure dependence of the Cu-plane Néel temperature *T*_N_ for *R*Ba_2_Cu_3_O_6+x_. The solid line is a fit and yields a slope of 23 K/kbar, indicating that this magnetic ordering is extremely sensitive to pressure [71].

For the cuprates, the central feature that controls many aspects of all the oxide materials is the strong copper-oxygen bonding, which results in a layered Cu-O crystal structure. In the undoped “parent” materials this strong bonding leads to an electrically insulating antiferromagnetic ground state [5]. The exchange interactions within the layers are much stronger than between the layers, and typically an order-of-magnitude more energetic than the lattice dynamics. The associated spin dynamics and magnetic ordering of the Cu ions is thus driven by this two-dimensional (2d) nature. This low dimensionality apparently makes the magnetic ordering temperature particularly sensitive to pressure as shown in Fig. 5 [71]. Here the Néel temperature for the Cu plane spins is plotted versus hydrostatic pressure. The ordering temperature increases with increasing pressure at the extraordinary rate of 23 K/kbar, where 1 kbar = 10^8^ Pa. In comparison, the rate of change of the superconducting transition temperature for YBa_2_Cu_3_O_7_ is more than two orders-of-magnitude smaller than for this magnetic transition.

With electronic doping, long range antiferromagnetic order for the S = 1/2 Cu spins typically is suppressed as metallic behavior and then superconductivity appears, but strong quantum spin fluctuations still persist in this regime. It is this large magnetic energy scale that is associated with the high superconducting transition temperature and exotic pairing. There is usually an interesting exception to the rule, however, and for the Cu spins a coexistence of magnetic order and superconductivity has recently been discovered in the single layer La_2_CuO_4+δ_ material, where the extra oxygen δ that dopes the system chemically orders in stages. The superconducting transition is sharp with an onset *T*_c_ = 42 K, the highest of any 2-1-4 system, while long range spin density wave magnetic order of the Cu moments is also observed in this material. The magnetic order is found to develop at the same transition temperature as the superconductivity, demonstrating that the magnetic order and superconductivity are inexorably linked [89].

**Fig. 6:**
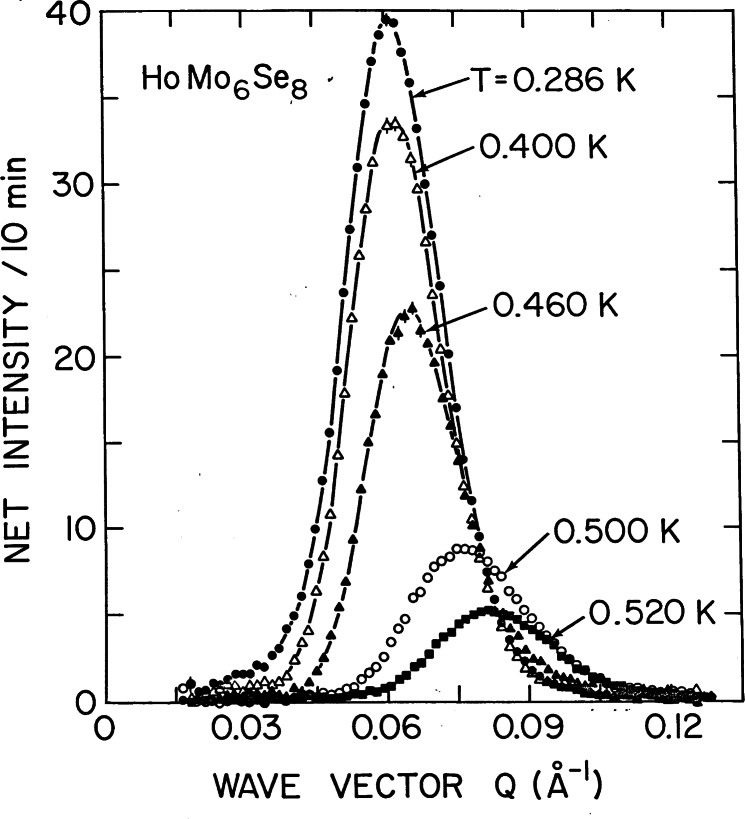
Temperature dependence of the magnetic scattering in the “ferromagnetic” superconductor HoMo_6_Se_8_. The compromise between the ferromagnetism, which would prefer to have the scattering peak at *Q* = 0, and the superconducting screening length (*λ*_L_ ≈10^3^ Å) results in the development of a long wavelength oscillatory magnetic state, which shifts to smaller wave vector as the amplitude of the magnetic order grows [35].

**Fig. 7:**
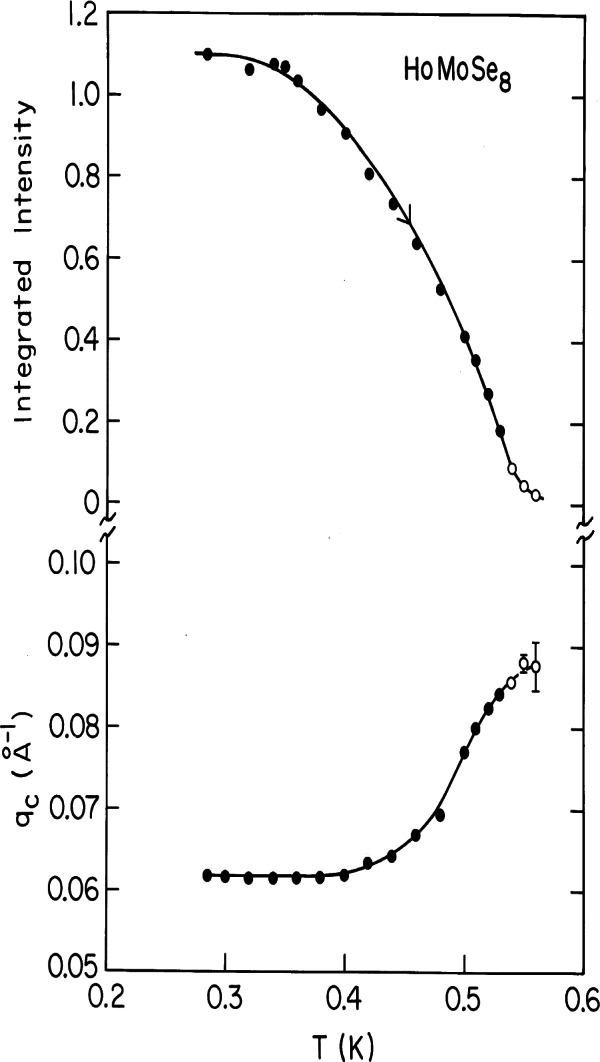
Temperature dependence of the integrated intensity (which is proportional to the square of the ordered moment) and the characteristic wave vector *q*_c_ for HoMo_6_Se_8_. The magnetic ordering develops at 0.53 K, well below the superconducting phase transition at 5.6 K. The solid curves are given by the theory, which incorporates the energetic balance between the magnetic order and the superconductivity [35].

In the rare and more interesting situation where the magnetic interactions are ferromagnetic, there is strong coupling to the superconducting state that originates from the internally generated magnetic field. Fig. 6 shows the magnetic scattering for HoMo_6_Se_8_, which becomes superconducting at 5.5 K, and then tries to order ferromagnetically at lower temperature [35]. A true ferromagnetic peak would be observed at *Q* = 0, but the competition between the superconducting order parameter and the ferromagnetic order gives rise to a long wavelength oscillatory magnetic state as shown in the figure. This is just a powder diffraction peak with a *d* spacing of ≈100 Å. Figure 7 shows that the strength of the scattering increases with decreasing temperature, while the wave vector decreases as the system tries to push closer to a ferromagnetic state (at *Q* = 0). However, the ferromagnetic energy is never large enough to quench the superconducting state, and the coexistence persists to low temperatures. For the related HoMo_6_S_8_ material the superconductivity is weaker (*T*_c_ = 1.8 K), and the material locks into ferromagnetism at low T, destroying the superconducting state [36]. In the ErNi_2_B_2_C system a small net magnetization develops at low temperatures, and the interesting situation is realized for the first time where a true net ferromagnetic order coexists with superconductivity [90]. Finally, we note that these magnetic superconductors have generated renewed interest very recently with the discovery of the mixed ruthenate-cuprate RuSr_2_GdCu_2_O_8_ system, where the Ru orders at 135 K with a ferromagnetic component in the magnetic structure, while superconductivity occurs at 30 K [91].

## 4. Magnetic Multilayers

**Fig. 8:**
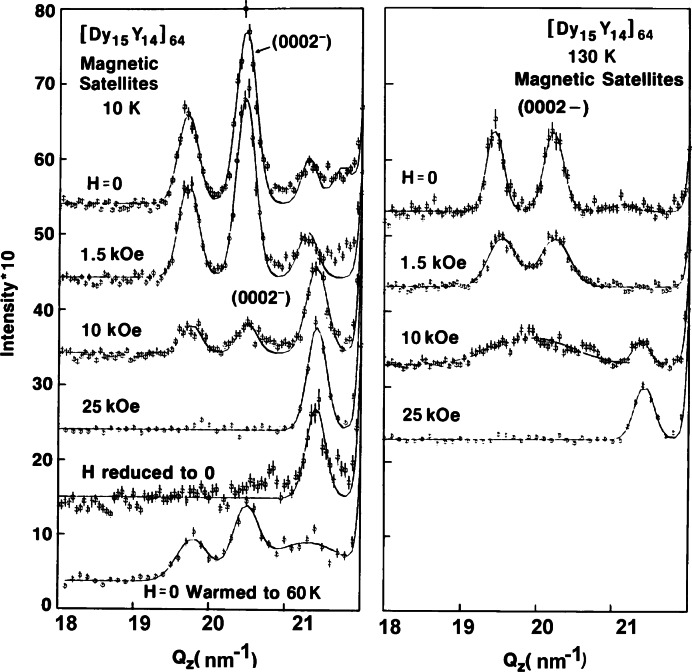
The left side shows the magnetic field dependence of the neutron diffraction peaks in a superlattice consisting of alternating layers of 15 atomic planes of dysprosium with 14 atomic planes of yttrium. The magnetic superlattice peaks disappear as the magnetic field converts the dysprosium helix to a ferromagnet. On the right side of the figure, at higher temperatures, the magnetic field breaks the coupling between helices in the separate dysprosium layers, leaving a broad peak centered at the dysprosium helix wave vector [43].

In recent years, composite and nanoscale structures have been at the center of many advances in materials’ properties and devices. Magnetic thin films and multilayers are examples of such structures and have been extensively studied at the NCNR. Many studies have focused on simple superlattices with magnetic and nonmagnetic layers designed to probe the interlayer magnetic coupling for materials with long-range (e.g., rare-earths and transition metals) and short-range (e.g., magnetic semiconductors and transition-metal oxides) exchange interactions [44]. Neutron diffraction measurements on rare-earth multilayers, for example, represent some of the very earliest work showing that exchange coupling information can be transmitted between magnetic layers through surprisingly thick nonmagnetic layers. Figure 8 shows neutron diffraction scans of the magnetic peaks in a film where 15 atomic planes of magnetic dysprosium are separated by 14 atomic planes of non-magnetic yttrium, and then this basic bilayer is repeated [43,45–46]. Multiple peaks are observed as a result of the superlattice structure of the film, and this implies that the dysprosium helical magnetic structure is coherent across multiple non-magnetic yttrium layers. The interlayer coupling can be readily controlled by modest magnetic fields, as shown in the figure. The right side of the figure shows how the breakdown of the coherence across the non-magnetic layers leads to the disappearance of the magnetic superlattice peaks. Interlayer coupling has also been observed and characterized in related superlattices composed of Dy/Lu [47] and Er/Y [48].

Studies of heavy rare-earth superlattices provided a basis for understanding the anomalous electronic and magnetic behavior of transition-metal multilayers which exhibit the giant magnetoresistive (GMR) effect. While it was generally assumed that the GMR is associated with an antiparallel alignment of the ferromagnetic layers across the nonmagnetic interlayers, neutron reflectivity studies of systems such as Co/Cu [49] and discontinuous Ni_80_Fe_20_/Ag multilayers [50] indicate that electron scattering from in-plane magnetic domains may also contribute to the effect. In another example, a neutron study [51] of (001) Fe(5.2 nm)/Cr (1.7 nm) superlattices showed that the low-field angle between the ferromagnetic Fe layers is 5°. In this system, the nature of the interlayer coupling in Fe/Cr multilayers is also correlated with the magnetic ordering of the Cr interlayers, which was characterized directly using high-angle neutron diffraction techniques [52]. The Fe layers exhibit non-collinear interlayer coupling above the *T*_N_ of Cr in samples with Cr layer thicknesses greater than 5 nm. The formation of the Cr spin density wave below *T*_N_ destroys this interlayer coupling [53]. Other recent research directions for transition-metal multilayers include studies of hydrogen loading in systems such as Fe/V [54], which emphasizes the importance of the Fermi surface in determining the interlayer coupling in GMR multilayers.

**Fig. 9:**
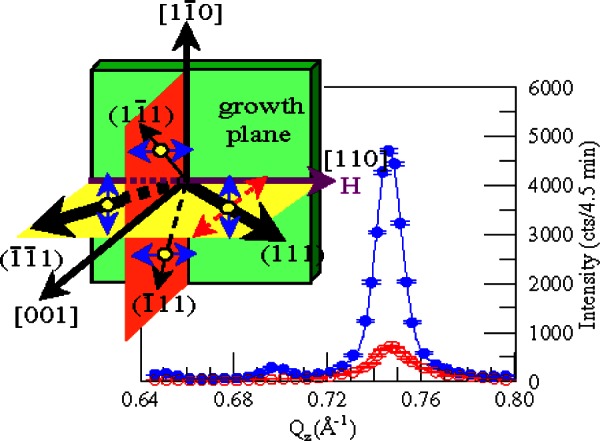
Non-spin-flip (solid) and spin-flip (open) cross sections for the (111) reflection scanned along the [001] growth axis direction. The data are for a [Fe_3_O_4_(100 Å)|CoO(30 Å]_50_ sample taken at 78 K in zero field after field cooling (*H* = 14 kOe) in the [110] direction. Inset illustrates the Co spin direction within each of the four {111} domains. More populated domains are indicated by thicker lines. For the (111) domain, the projections sensed by the non-spin-flip (solid arrow) and spin-flip (dotted arrow) cross sections are shown [55].

Similar exchange coupling has been observed in transition-metal oxide multilayers. Early studies of transition-metal oxides focused on multilayers composed of a ferrimagnet and an antiferromagnet, such as Fe_3_O_4_/CoO [55–56] and Fe_3_O_4_/NiO [57–58] or of alternating antiferromagnets, such as CoO/NiO [59]. While the multilayers retain the spin structures of their bulk constituents, the composite magnetic behavior is strongly influenced by local coupling at the interfaces. Some of these materials are now being used in a variety of applications such as high-sensitivity magnetic sensors and read/write heads [44]. For some of these applications, an antiferromagnetic film with a large anisotropy is grown on top of a ferromagnet, producing an exchange-biasing (i.e., a weak uni-directional anisotropy). The research at the NCNR has lead to a better understanding of the magnetic interactions responsible for this exchange-bias phenomenon. For example, high-angle diffraction studies of Fe_3_O_4_/NiO superlattices reveal that the exchange biasing is correlated with “frozen” magnetic domain walls within the antiferromagnetic NiO layers [58]. In related investigations of Fe_3_O_4_/CoO superlattices [55] it was demonstrated that the ferrimagnetic Fe_3_O_4_ and the antiferromagnetic CoO moments are aligned at 90° relative to each other due to the interlayer exchange coupling. Figure 9 shows a polarized neutron scan through the (111) antiferromagnetic reflection for a [Fe_3_O_4_(100 Å)|CoO(30 Å)]_50_ superlattice after cooling in a large field. The non-spin-flip intensity is substantially larger than the spin-flip direction indicating that the antiferromagnetic spins are preferentially aligned perpendicular to the applied field direction and thus perpendicular to the Fe_3_O_4_ moments. The spin structure determined from the neutron studies is shown in the inset of the figure. This experiment emphasizes the importance of the details of the antiferromagnetic structure for realistic models of exchange biasing.

## 5. Future

In recent years the new suite of cold neutron instrumentation has developed into the best facilities available in the U.S., and these new world-class neutron spectrometers have dramatically improved our measurement capability for exploring the properties of magnetic materials. Presently we are developing a new suite of thermal neutron instrumentation that will be unparalleled in this country, and we anticipate that these new instruments will produce an equally important impact on future investigations of magnetic phenomena.

One of the advantages of working at a neutron facility with a suite of modern instruments is that one has the ability to explore a wide range of phenomena, from domain structures, ferrofluids, and magnetically active bio-organisms with SANS, to multilayer magnets with reflectometry, to magnetic diffraction studies as a function of temperature, pressure, and applied magnetic and electric fields. Magnetic neutron scattering presently plays a dominant role in addressing these kinds of problems, and this will no doubt continue for many years to come.
